# Measures of Brain Connectivity and Cognition by Sex in US Children

**DOI:** 10.1001/jamanetworkopen.2023.0157

**Published:** 2023-02-21

**Authors:** Dardo Tomasi, Nora D. Volkow

**Affiliations:** 1 Laboratory of Neuroimaging, National Institute on Alcohol Abuse and Alcoholism, Bethesda, Maryland; 2 National Institute on Drug Abuse, Bethesda, Maryland

## Abstract

**Importance:**

The neurobiological underpinnings underlying sex differences in cognition during adolescence are largely unknown.

**Objective:**

To examine sex differences in brain circuitry and their association with cognitive performance in US children.

**Design, Setting, and Participants:**

This cross-sectional study analyzed behavioral and imaging data from 9- to 11-year-old children from the Adolescent Brain Cognitive Development (ABCD) study between August 2017 and November 2018. The ABCD study is an open-science, multisite study following up more than 11 800 youths into early adulthood for 10 years with annual laboratory-based assessments and biennial magnetic resonance imaging (MRI). The selection of ABCD study children for the current analysis was based on the availability of functional and structural MRI data sets in ABCD Brain Imaging Data Structure Community Collection format. Five hundred and sixty participants who had excessive level of head motion (>50% of time points with framewise displacement >0.5 mm) during resting-state functional MRI were excluded from the analyses. Data were analyzed between January and August 2022.

**Main Outcomes and Measures:**

The main outcomes were the sex differences in (A) global functional connectivity density at rest and (B) mean water diffusivity (MD) and (C) the correlation of these metrics with total cognitive scores.

**Results:**

A total of 8961 children (4604 boys and 4357 girls; mean [SD] age, 9.92 [0.62] years) were included in this analysis. Girls had higher functional connectivity density in default mode network hubs than boys, predominantly in the posterior cingulate cortex (Cohen *d* = −0.36), and lower MD and transverse diffusivity, predominantly in the superior corticostriatal white matter bundle (Cohen *d* = 0.3). Age-corrected fluid and total composite scores were higher for girls than for boys (Cohen *d* = −0.08 [fluid] and −0.04 [total]; *P* = 2.7 × 10^−5^). Although total mean (SD) brain volume (1260 [104] mL in boys and 1160 [95] mL in girls; *t* = 50; Cohen *d* = 1.0; *df* = 8738) and the proportion of white matter (*d* = 0.4) were larger for boys than for girls, the proportion of gray matter was larger for girls than for boys (*d* = −0.3; *P* = 2.2 × 10^−16^).

**Conclusions and Relevance:**

The findings of this cross-sectional study on sex differences in brain connectivity and cognition are relevant to the future creation of brain developmental trajectory charts to monitor for deviations associated with impairments in cognition or behavior, including those due to psychiatric or neurological disorders. They could also serve as a framework for studies investigating the differential contribution of biological vs social or cultural factors in the neurodevelopmental trajectories of girls and boys.

## Key Points

**Question:**

Do differences in cognition reflect differences in brain connectivity between boys and girls in late childhood?

**Findings:**

In this cross-sectional neuroimaging study of 8961 children aged 9 to 11 years, compared with boys, girls had higher global functional connectivity density in posterior cingulate cortex that increased in proportion to cognitive performance and lower mean diffusivity in superior corticostriatal white matter bundles, which decreased in proportion to increased cognitive performance. In mediation analyses, global functional connectivity density and mean diffusivity fully mediated the sex differences in cognitive performance.

**Meaning:**

These findings suggest that the sex differences in cognitive performance and brain connectivity in this study likely reflect faster brain maturation in girls than boys.

## Teaser

This cross-sectional study of US children aged 9 to 11 years assesses functional connectivity hubs displaying prominent sex differences and the association between global functional connectivity density and total cognitive scores.

## Introduction

Sex-related variations in brain structure and function may underly differences in cognition and behavior and in the prevalence of psychiatric disorders (ie, anxiety, depression, autism spectrum disorder, and attention-deficit/hyperactivity disorder) between men and women.^1,2,3,4,5,6,7^ Both biological and social or cultural factors are likely to drive these sex-related variations. Overall, men have larger total brain volume (BV) than women,^8,9^ and although an association between BV and general intelligence has been reported,^10^ meta-analyses have found similar cognitive performances between the sexes, with women having slightly better verbal skills and faster perceptual speed than men^11^ and men having slightly better spatial skills than women.^12^

Brain maturation is faster in women than men.^13,14,15^ Although by the age of 6 years the brain has already reached 90% of its adult size, the gray matter (GM) and white matter (WM) volumes continue to change throughout adolescence. Brain development during childhood and adolescence^16,17^ is characterized by a progressive WM volume increase with age, which is faster for girls^18^; regressive GM pruning, which is faster for boys^19^; and gradual increase in GM density, which is faster in girls, such that girls have higher GM density than boys across the entire brain after 8 years of age.^20^

Sex differences in brain anatomy are likely to be associated with differences in functional and structural connectivity between men and women because changes in brain structure are coupled with changes in brain function during development.^21^ In the resting state, women have higher homotopic connectivity in dorsolateral prefrontal cortex^22^ and stronger functional connectivity density (FCD) in hubs of the default mode network (DMN) than adult men.^23,24^ During development, boys have stronger between-module connectivity than girls, but girls have stronger within-module connectivity than boys.^25^ However, the reproducibility of these findings is uncertain, and little is known about the sex differences in structural connectivity or the association between structural and functional connectivity and cognitive performance.

The current cross-sectional study aimed to assess sex differences in structural and functional connectivity and in total cognition in 9- to 11-year-old children (n = 8961) from the Adolescent Brain Cognitive Development (ABCD) study. We hypothesized that the large ABCD cohort would allow us to reveal significant and reproducible sex differences in cognition and structural and functional connectivities despite their intrinsic small effect sizes. Our overarching hypothesis was that due to their earlier maturation, girls would have higher cognitive performance and stronger structural and functional connectivities than boys and that higher cognitive performance would be associated with increased functional connectivity.

We used data-driven FCD mapping^26^ to pinpoint functional connectivity hubs displaying prominent sex differences and to assess the association between global FCD (gFCD) and total cognitive scores. We hypothesized that the posterior DMN hub (posterior cingulate cortex [PCC]^27^), which is a central node, would reveal significant associations for sex and cognition scores. In addition, we used region-of-interest (ROI) analysis to assess the associations of sex and total cognition with structural connectivity using WM diffusion metrics and their association with the functional connectivity metrics. Because of the sex differences in maturation, we hypothesized that higher cognition scores would be associated with higher anisotropic diffusion, which would be higher in girls than in boys. Furthermore, using causal mediation analysis (CMA), we tested the hypothesis that structural and functional connectivities would mediate the association of sex with total cognition.

## Methods

### Participants

In this cross-sectional study, we analyzed deidentified behavioral and imaging data from 9521 children aged 9 to 11 years in the ABCD 2.0 data release^28^ for whom resting-state functional magnetic resonance imaging (fMRI) data in CIFTI format were available. A total of 560 participants with excessive level of head motion during resting-state fMRI (>50% of time points with framewise displacement >0.5 mm) were excluded from the analyses. The final sample for the studies on sex differences in cortical thickness (CT) and resting-state functional connectivity included 8961 children (4357 girls and 4604 boys). The ABCD study, which is an ongoing, 10-year, longitudinal study initiated in 2017, obtained centralized institutional review board approval from the University of California, San Diego, and local institutional review board approval at 21 data collection sites across the US.^29^ Children provided written assent for their participation. Written informed consent was also provided by parents. The present study was deemed exempt from the need for informed consent by the institutional review board at the National Institutes of Health and followed the Strengthening the Reporting of Observational Studies in Epidemiology (STROBE) guideline.

### MRI Data

The MRI acquisition protocols are described in the eMethods in Supplement 1 and elsewhere.^30,31^ We used the ABCD Brain Imaging Data Structure Community Collection (ABCC),^32^ which encompasses MRI and resting-state fMRI data in Human Connectome Project–compatible format (eg, CIFTI)^33^ from 10 038 children who have passed quality assurance of the National Institute of Mental Health Data Archive.^34^ To minimize unwanted variability from differences in MRI instruments, ABCD Brain Imaging Data Structure adopted and modified the Human Connectome Project pipeline to accommodate data from GE, Phillips, and Siemens scanners (eMethods in Supplement 1). Tabulated diffusion MRI (dMRI), including fractional anisotropy (FA), mean diffusivity (MD), longitudinal diffusivity, transverse diffusivity (tD), fiber track volume (FTV), and CT data of the ABCD study were downloaded from the National Institute of Mental Health Data Archive website.^35^ Morphometric FreeSurfer analysis and dMRI data preprocessing steps of the ABCD study are described in the eMethods in Supplement 1 and elsewhere.^31^

### Reproducibility

The large ABCC sample allowed us to split participants into 3 independent, demographically matched subsamples: discovery (n = 4405 [2181 girls]), replication (n = 4334 [2066 girls]), and normality (n = 222 [110 girls]) using ABCC’s matched group status.^34^ The ABCC matching status was based on 9 sociodemographic factors that can impact brain development (site, age, sex, ethnicity, grade, highest level of parental education, combined family income, exposure to anesthesia, and handedness). Significant differences were found in age, race, BV, framewise displacement, and fluid and crystallized composite scores between the 4247 girls and 4492 boys (Table), which we controlled for in statistical analyses. Because FA and MD ABCD metrics are highly variable across MRI scanners,^31^ the study of sex differences in structural connectivity was restricted to 5797 participants (2779 girls and 3018 boys) who underwent MRI on Siemens scanners.

**Table.** Characteristics of the Subsamples

Characteristic	Reproducibility subsample			Sex subsample		
	Discovery (n = 4405)	Replication (n = 4334)	*P* value	Girls (n = 4247)	Boys (n = 4492)	*P* value
Male-female ratio	1.02	1.1	.08	1.06	0.98	.08
Age, mean (SD), mo	119.08 (7.47)	119.45 (7.42)	.02	119.10 (7.42)	119.50 (7.48)	.03
Race, No. (%)						
African American	590 (13.4)	579 (13.4)	.94	613 (14.4)	556 (12.4)	.02
Asian	81 (1.8)	80 (1.8)		89 (2.1)	72 (1.6)	
Hispanic	849 (19.3)	832 (19.2)		820 (19.3)	861 (19.2)	
White	2439 (55.4)	2378 (54.9)		2287 (53.8)	2530 (52.3)	
Other^a^	446 (10.6)	465 (10.7)		438 (10.3)	473 (10.5)	
Brain volume, mean (SD), mL	1207.71 (112.60)	1213.07 (114.23)	.03	1154.73 (94.58)	1262.66 (104.46)	<.001
Framewise displacement, mean (SD), mm	0.117 (0.041)	0.116 (0.041)	.15	0.113 (0.040)	0.121 (0.042)	<.001
Scanner, No. (%)						
Siemens	2872 (65.2)	2925 (67.5)	.04	2779 (65.4)	3018 (67.2)	.17
GE	1026 (23.3)	912 (21.0)		958 (22.6)	980 (21.8)	
Phillips	507 (11.5)	497 (11.5)		510 (12.0)	494 (11.0)	
Family income bracket, mean (SD)	7.36 (2.32)	7.35 (2.31)	.85	7.36 (2.30)	7.35 (2.33)	.81
Fluid composite, mean (SD)	92.11 (10.04)	92.21 (10.01)	.66	92.51 (9.88)	91.82 (10.15)	.002
Crystalized composite, mean (SD)	86.75 (6.54)	86.73 (6.60)	.89	86.56 (6.46)	86.90 (6.66)	.02
Total composite, mean (SD)	86.80 (8.37)	86.82 (8.40)	.89	86.92 (8.26)	86.70 (8.49)	.25

^a^ Other includes Native American, Pacific Islander, unknown, or more than 1 race.

### Statistical Analysis

The strength of the gFCD^24^ was mapped from individual CIFTI time series with 91 282 gray ordinates^33^ and a maximum of 1520 time points (20 minutes) using a correlation threshold of *R*>0.6 in the Biowulf cluster. For the independent normality subsample, we confirmed the normal distribution of gFCD using the Shapiro-Wilk normality test (W > 0.98; *P* = .07). Before statistical analysis, we regressed out associations of brain size and head motion across participants using linear regression, independently for boys and girls, and we also removed unwanted associations with race using grand mean scaling (race, which was reported by the parent or caregiver as African American, Asian, Hispanic, White, or other, was included in the study due to the confounding nature of these variables in the ABCD data sets) . Because morphometric and functional ABCD metrics vary significantly across MRI manufacturers,^31^ we similarly removed site- and scanner-specific differences, independently for boys and girls. Then, factorial analyses of covariance (ANCOVAs) were conducted in Matlab, version 2017b (Mathworks Inc) (CIFTI data) or R, version 4.0 (R Foundation for Statistical Computing) (ROI data), independently for the discovery and replication subsamples, to study the main associations of sex, age, and the total cognitive composite with dependent variables (CT, gFCD, FA, MD, longitudinal diffusivity, tD, and FTV); we tested for interactions between sex and total cognition on ROI mean values for the dependent variables in the PCC or the superior corticostriatal (SCS) WM bundle with R software. A false discovery rate (FDR)–corrected *P* < .05 was used to correct for multiple comparisons across 91 282 gray ordinates in the CIFTI data. A Bonferroni correction for 42 major WM tracts in the AtlasTrack was used to correct dMRI results for multiple comparisons. To quantify sex differences, we used Cohen *d* (the difference between mean dependent variable values for boys and girls divided by the pooled SD). Partial η^2^ was used in conjunction with ANCOVA to estimate the effect sizes of categorical and continuous factors.

In CMAs, the mediation R package^36^ was used to estimate causal mediation effects. One thousand bootstrapping samples and a heteroskedasticity-consistent estimator for the covariance matrix were used to estimate the mean direct and causal mediation effects. A Bonferroni correction for 3 metrics (gFCD, MD, and CT) was used to correct the CMA results for multiple comparisons. A 2-sided *P* < .05 corrected for multiple comparisons was considered statistically significant.

## Results

### Cognitive Performance and Brain Volume

**Figure 1.**
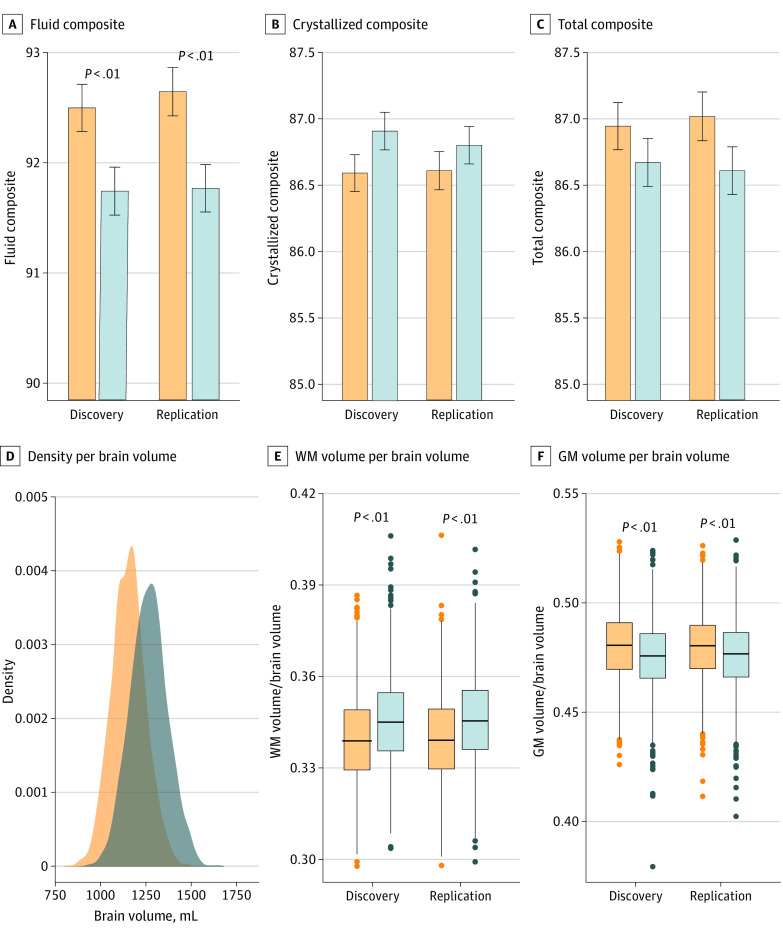
Sex Differences in Cognition and Brain Anatomy Bar plots showing the reproducibility of sex differences in fluid, crystallized, and total cognitive composites in the discovery (n = 2181 girls [yellow] and 2224 boys [blue]) and replication (2066 girls and 2268 boys) subsamples (top). Density plot showing the distribution of brain volume across 4247 girls and 4492 boys and boxplots showing the reproducibility of sex differences in the proportions of white matter (WM) and gray matter (GM) in the discovery and replication subsamples (bottom).

A total of 8961 children (4604 boys and 4357 girls; mean [SD] age, 9.92 [0.62] years; 1169 [13.4%] African American, 161 [1.9%] Asian, 1681 [19.2%] Hispanic, 4817 [55.1%] White, and 911 [10.4%] other race or ethnicity) were included in this analysis (Table). Age-corrected fluid and total composite scores were higher for girls than for boys (Cohen *d* = −0.08 [fluid] or −0.04 [total]; *P* = 2.7 × 10^−5^) (Figure 1A). The association of age with uncorrected composite scores was not significant. Although total mean (SD) BV (1260 [104] mL in boys and 1160 [95] mL in girls; *t* = 50; Cohen *d* = 1.0; *df* = 8738) and the proportion of WM (d = 0.4) were larger for boys than for girls, the proportion of GM was larger for girls than for boys (*d* = −0.3; *P* = 2.2 × 10^−16^) (Figure 1B). The associations of age with BV as well as GM and WM proportions were not significant.

### Functional Connectivity

**Figure 2.**
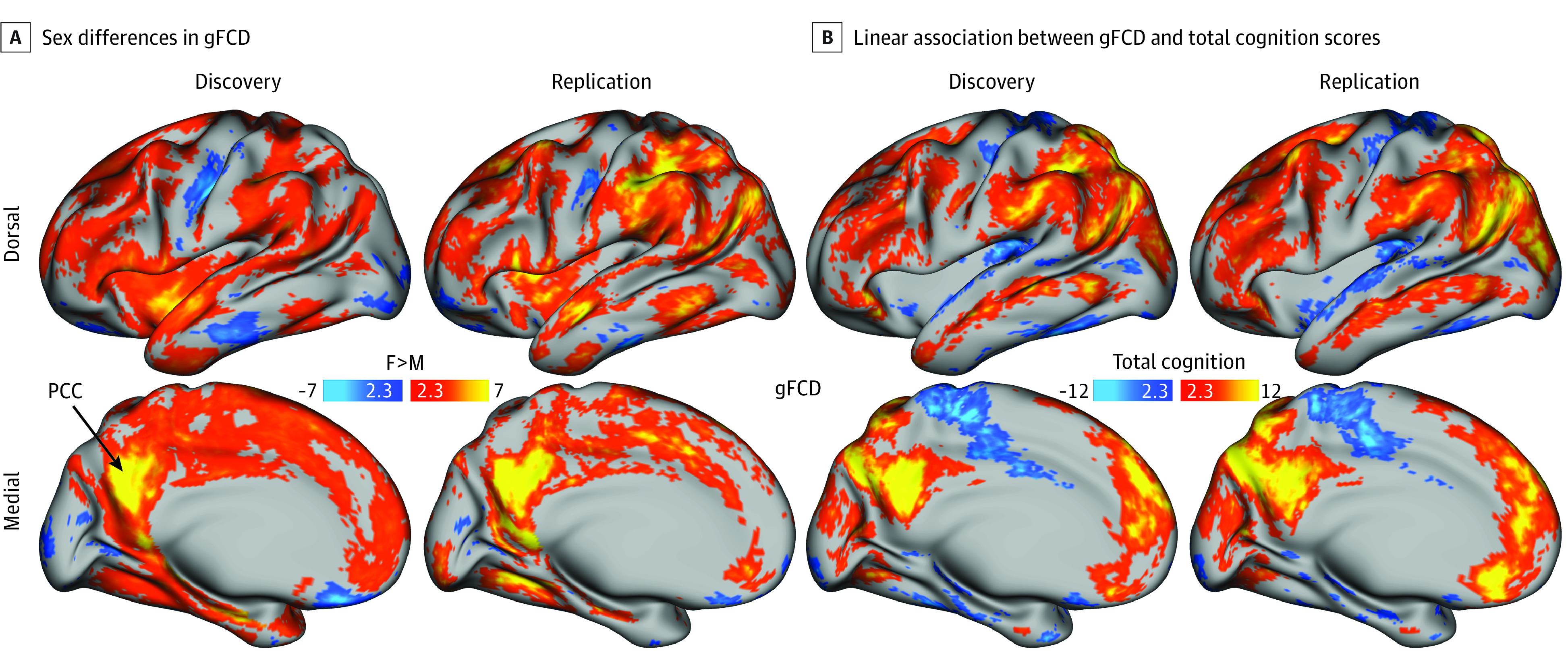
Sex Differences in Global Functional Connectivity Density (gFCD) Statistical significance (*t* score) for the sex differences in gFCD (A) and the linear association between gFCD and total cognition scores (B) in the discovery (2181 girls and 2224 boys) and replication (2066 girls and 2268 boys) subsamples, rendered on left dorsal and medial brain surfaces. A false discovery rate–corrected *P* < .05 was used to control for multiple comparisons across 91 282 gray ordinates. Statistical model was analysis of covariance. The arrow points to the location of the posterior cingulate cortex (PCC).

Vertex-wise ANCOVA demonstrated a highly reproducible bilateral pattern of sex differences in gFCD (Figure 2A). Compared with boys, girls exhibited higher gFCD in multiple cortical and subcortical regions, which was most prominent in the PCC (a 10-mm radius ROI centered at vertex 11806 in the left cerebral hemisphere; Cohen *d* = −0.36) (eFigure 1A in Supplement 1), and lower gFCD in the somatomotor cortex, visual areas, medial orbitofrontal cortex, and inferior temporal gyrus. The reproducibility of this pattern in the discovery and replication subsamples was high (*R* = 0.54, correlation of mean *t* score maps across 91 282 gray ordinates) (eFigure 2F in Supplement 1). The bilateral associations of total cognition with gFCD were also highly reproducible (Figure 2B). Specifically, increased total composite scores were associated across individuals with higher gFCD in PCC (eFigure 1B in Supplement 1) and other DMN regions, as well as dorsolateral prefrontal, occipital, and parietal cortices, middle temporal gyrus, and posterior cerebellar hubs. Higher cognition scores were also associated with lower gFCD in the somatomotor cortex, middle temporal visual area (V5), superior temporal gyrus, and most subcortical regions. The reproducibility of this pattern in the discovery and replication subsamples was high (*R* = 0.96). The mean (SD) patterns of main associations of sex and total cognition with gFCD had significant overlap (36% [1%]). The sex difference (*d* = −0.37) explained 4% and total cognition explained 2% of the variance in gFCD in PCC (eTable in Supplement 1). A significant association of age with gFCD was not found.

### Cortical Thickness

Compared with boys, girls had thicker anterior cingulum and parahippocampal gyrus and thinner occipital cortex. Higher total cognitive composite scores were associated with lower CT, predominately in the medial prefrontal cortex and insula (eFigure 3 in Supplement 1). The reproducibility of these patterns in the discovery and replication subsamples was high (*R* > 0.64), but the mean (SD) overlap between the patterns of sex differences and the association of CT with cognition was modest (9% [1%]). The effect size of the sex difference in CT in the PCC ROI was very small (*d* = 0.01). The effect sizes of age on CT were not significant.

### Structural Connectivity

**Figure 3.**
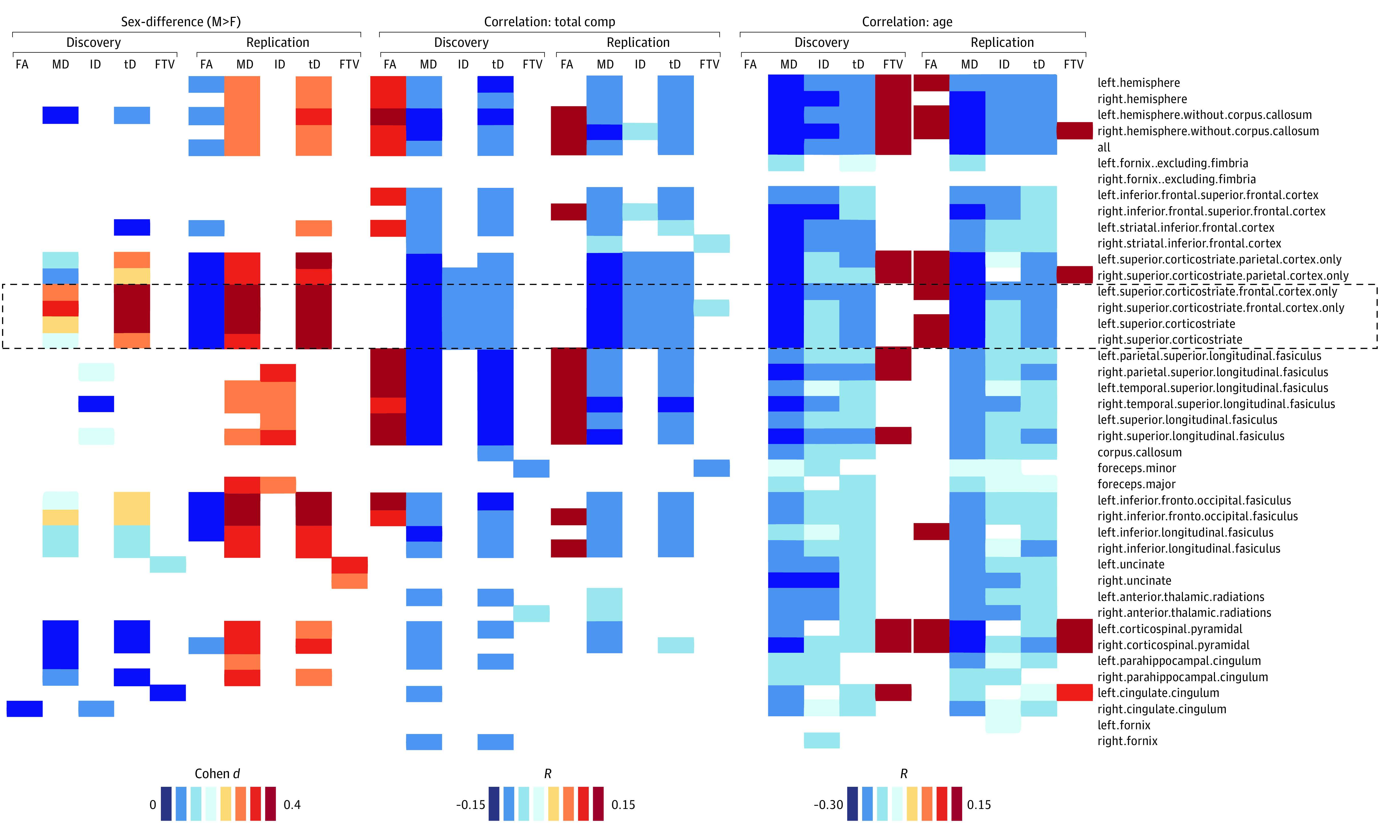
Effects of Sex, Total Cognition, and Age on Diffusion Magnetic Resonance Imaging (dMRI) Measures Statistically significant sex differences (Cohen *d*) in brain volume–corrected fractional anisotropy (FA), mean diffusivity (MD), longitudinal diffusivity (lD), transverse diffusivity (tD), and fiber track volume (FTV; left) and significant Pearson correlations of these white matter diffusion metrics with the total cognition composite (middle) and age (right) for 42 major white matter fiber bundles in the discovery (1432 girls and 1440 boys) and replication (1347 girls and 1578 boys) subsamples. Data were collected with Siemens MRI scanners. Statistical model was Bonferroni-corrected analysis of covariance.

**Figure 4.**
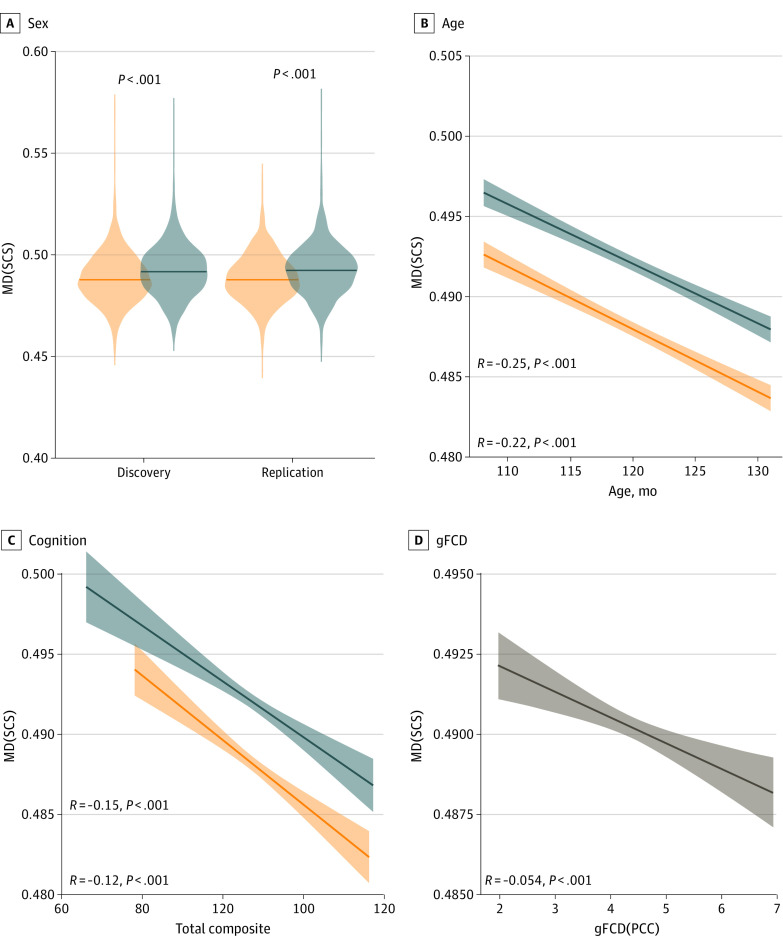
Associations of Mean Diffusivity (MD) With Age, Cognition, and Global Functional Connectivity Density (gFCD) Reproducibility of the sex difference in MD (brain volume corrected) in the right superior corticostriatal (SCS) bundle and its associations with age and the total composite independently for 2779 girls (orange) and 3018 boys (blue) and the mean gFCD in the posterior cingulate cortex (PCC) across all 5797 individuals.

There were no reproducible sex differences in FA or FTV. In contrast, several major WM fiber bundles demonstrated higher MD and tD for boys than girls (Figure 3). Specifically, the bilateral SCS and inferior corticostriatal bundles demonstrated small (*d* = 0.2-0.3) but reproducible and statistically significant sex differences in MD and tD, such that girls had lower MD and tD than boys (Figure 3 and Figure 4). Higher MD in the right SCS bundle was associated with lower total cognitive composite scores in the discovery and replication subsamples in both boys and girls (Figure 3 and Figure 4). The sex difference explained 2% of the variance in MD and 1% in tD, and the total cognitive composite explained 2% of the variance in MD or tD on these bundles (eTable in Supplement 1); MD was highly correlated with tD (*R* = 0.77) but less so with FA (*R* = −0.36) across children. Age was associated with increased FA and decreased MD in both boys and girls, despite the restricted age sample (Figure 3 and Figure 4). Lower MD in SCS bundles was associated with higher gFCD, independently in boys and girls (Figure 4). No significant age × sex interactions were found on dMRI metrics.

### Mediation Analysis

Model 1 of the CMA (eFigure 4 in Supplement 1) demonstrated the full mediation of the functional and structural connectivity metrics (mean values of gFCD for PCC and mean values of MD for SCS bundles) on the sex differences in the total cognitive composite scores, whereas the mean CT in the PCC ROI did not mediate the sex differences in total cognitive composite scores. Model 2 of the CMA did not reveal mediation effects of total cognitive composite scores on sex differences in gFCD or MD measures.

## Discussion

In this cross-sectional study, we uncovered robust sex differences and associations of total cognition with structural and functional connectivity metrics in a large cohort of US children. The current study revealed significant associations of sex and total cognition with gFCD during the resting state, which were stronger for girls than for boys and increased in association with improved cognitive performance, whereas MD was higher for boys than girls and was negatively associated with cognition. Fractional anisotropy was associated with cognitive performance but not with sex. These findings were highly reproducible in the discovery and replication subsamples. Furthermore, we found that gFCD and MD, but not CT, fully mediated the sex differences in cognitive performance.

We found higher gFCD in DMN hubs for girls than boys, which is consistent with prior findings of stronger DMN functional connectivity for adult women than men^23,27,37^ and suggests that girls have stronger DMN connectivity than boys. The PCC, a metabolically demanding hub^38^ that is functionally connected to other DMN regions,^27^ housed the largest sex differences in functional connectivity. This finding is in line with prior findings of DMN hypoconnectivity in neurodevelopmental disorders, such as autism spectrum disorder and attention-deficit/hyperactivity disorder,^39,40^ which are 2 to 4 times more common among boys than girls.^41^ The lack of significant sex effects on CT indicates that sex differences in brain connectivity in the current study were not confounded by sex differences in CT.

Although we did not find sex differences in FA, we documented lower MD and tD in SCS bundles for girls than boys, which is consistent with findings from prior studies that reported a more distributed structural connectivity in frontal, parietal, and temporal lobes in women than in men^42^ and earlier WM development in women compared with men.^43^ Our findings of significant increases in FA and decreases in MD with age are consistent with the development of WM bundles during childhood^43^ and support the delayed maturation of brain structure in men compared with women.^15,44^ The lack of significant sex × age interaction effects on the other dMRI metrics likely reflects the narrow age range in the current study.

The brain volumes of boys (1260 mL) and girls (1160 mL) in the current study corresponded well with those of healthy adult men (1260 mL) and women (1130 mL) of European ancestry.^45^ The smaller sex differences in brain volume for children in the current study (*d* = 1.00) compared with adults (*d* = 1.26)^46^ could be partially explained by the larger variability in brain volume for children in the ABCD study (pooled SD, 74 mL) compared with adults (66 mL^46^) and may also reflect the earlier brain maturation in girls.^15,47^ Because we did not find differences in CT in the PCC, our findings regarding sex differences in brain structural and functional connectivity were unlikely to be confounded by sex differences in GM maturation,^15,19^ which could be associated with changes in brain function.^21^

Cognitive performance improved with stronger gFCD, consistent with the notion that increased functional connectivity is associated with intelligence,^48,49^ particularly in children.^50^ Prior fMRI studies in adults have found associations between sex and fMRI activation^51^ and that fluid intelligence increased with the coupling between dorsal attention network regions and with the negative coupling between DMN and dorsal attention network regions,^52^ consistent with our findings in children. Strikingly, there was significant overlap between the regional effects of sex and cognitive performance on gFCD, such that the girls’ cognitive advantage was supported by their gFCD and by the association between gFCD and cognitive performance.

We also found that cognitive performance decreased with MD in striatocortical WM fibers. These findings are overall consistent with the role of structural connectivity in intelligence.^53,54^ As in prior dMRI studies,^55^ we found positive associations with cognitive performance for FA and negative ones for MD and tD. In addition, we found positive associations with age for FA and negative ones for MD and tD, which suggests that structural connectivity strengthens during the maturation of cognitive abilities in late childhood. The lack of significant age × cognition interactions in the current study, however, does not support the development of a negative association between cognitive abilities and brain connectivity during childhood, as documented for children and adolescent boys.^53,54^ We found higher fluid cognition scores for girls than boys, a sex difference in cognitive abilities that is likely associated with the earlier brain maturation of girls.^15,56,57,58^ Using CMA, we found that functional and structural connectivity metrics fully mediated the association of sex and total cognition, perhaps due to sex differences in the trajectories of brain development.

### Limitations

Limitations include the cross-sectional nature of the current study, which does not allow us to confer causality. However, the longitudinal nature of the ABCD study will enable future studies to test whether increased functional and structural connectivity, as neurodevelopment proceeds, is associated with improvements in cognitive performance.

## Conclusions

In this cross-sectional study, we document reproducible moderate sex differences in brain connectivity and their associations with sex differences in cognition in preadolescent US children. Although there are robust sex differences in brain connectivity that are likely to reflect in part earlier brain development in girls than boys due to sex-associated differences in gene transcription patterns and the effects of gonadal hormones in the brain, social and cultural factors are also likely to contribute.^59^ Notwithstanding the robustness of the sex differences in brain connectivity and their association with cognition, together they explain a relatively small amount of the overall variance in brain connectivity. Meanwhile, research has started to document how social environmental exposures^60^ as well as other social determinants of health^61^ influence brain development, highlighting the potential value that standardized graphs of normative brain development can have to help screen for psychiatric neurodevelopmental disorders and to guide personalized therapeutic interventions.

## Notes

Supplement 1. **eMethods.** Supplemental Methods **eFigure 1.** Global Functional Connectivity Density (gFCD) In the Posterior Cingulate Cortex (PCC): Reproducibility and Association With Cognition **eFigure 2.** Reproducibility of Sex Differences in gFCD **eFigure 3.** Effects of Sex and Total Cognition on Cortical Thickness **eFigure 4.** Causal Mediation Analysis (CMA) **eTable.** Effects of Sex, Total Composite Score, and Testosterone on Average Values of Global Functional Connectivity Density (gFCD) and Cortical Thickness (CT) in the Posterior Cingulum Seed, and on Average Values of Mean Diffusivity (MD) in the Right Cortico-Striatal Fiber Bundle
eReferences

Supplement 2.
Data Sharing Statement
