# Supplementary material for: Measures of Brain Connectivity and Cognition by Sex in US Children
Source: JAMA Netw Open. 2023 Feb 21;6(2):e230157. doi: 10.1001/jamanetworkopen.2023.0157 (PMC9945095; doi:10.1001/jamanetworkopen.2023.0157)
Supplement: Supplement 1. — eMethods. Supplemental Methods eFigure 1. Global Functional Connectivity Density (gFCD) In the Posterior Cingulate Cortex (PCC): Reproducibility and Association With Cognition eFigure 2. Reproducibility of Sex Differences in gFCD eFigure 3. Effects of Sex and Total Cognition on Cortical Thickness eFigure 4. Causal Mediation Analysis (CMA) eTable. Effects of Sex, Total Composite Score, and Testosterone on Average Values of Global Functional Connectivity Density (gFCD) and Cortical Thickness (CT) in the Posterior Cingulum Seed, and on Average Values of Mean Diffusivity (MD) in the Right Cortico-Striatal Fiber Bundle eReferences [file jamanetwopen-e230157-s001.pdf]

Supplementary Online Content

Tomasi D, Volkow ND. Measures of brain connectivity and cognition by sex in US children. *JAMA Netw Open*. 2023;6(2):e230157. doi:10.1001/jamanetworkopen.2023.0157

eMethods. Supplemental Methods
eFigure 1. Global Functional Connectivity Density (gFCD) In the Posterior Cingulate Cortex (PCC): Reproducibility and Association With Cognition
eFigure 2. Reproducibility of Sex Differences in gFCD
eFigure 3. Effects of Sex and Total Cognition on Cortical Thickness
eFigure 4. Causal Mediation Analysis (CMA)
eTable. Effects of Sex, Total Composite Score, and Testosterone on Average Values of Global Functional Connectivity Density (gFCD) and Cortical Thickness (CT) in the Posterior Cingulum Seed, and on Average Values of Mean Diffusivity (MD) in the Right Cortico-Striatal Fiber Bundle
eReferences

This supplementary material has been provided by the authors to give readers additional information about their work.

© 2023 Tomasi D et al. *JAMA Network Open.*

eMethods. Supplemental Methods

Recruitment. Deatails on recruitment strategies and inclusion and exclusion criteria for the ABCD study have been published and are available on the ABCD website (https://abcdstudy.org/scientists/protocols/). Briefly, the ABCD used probability sampling of U.S. schools within 21 catchment areas (geographical areas centered on schools within 50 miles of the research institution) as the primary method for contacting and recruiting eligible children and their parents.[1] Recruitment materials and electronic copies were provided to the families. Interested families completed a brief telephone screening and, if eligible, were enrolled and scheduled for the baseline assessment, which occurred at the research centers. Guardians and children were reimbursed for their participation.[1] Recruitment closely represented demographic variables (sex, race, ethnicity, parental marital status and education, and income) of the general US population.[2]

Inclusion and Exclusion criteria: Children were not excluded unless they had severe psychiatric or neurological disorders or suffered from severe medical conditions. Specifically, exclusion criteria included common MRI contraindications, inability to understand or speak English fluently, uncorrected vision, hearing or sensorimotor impairments, a history of major neurological disorders, gestational age <28 weeks, birth weight <1,200 g, birth complications that resulted in hospitalization for more than 1 month, current diagnosis of schizophrenia, moderate or severe autism spectrum disorder, a history of traumatic brain injury or unwillingness to complete assessments.[3,4] ADHD children with or without medications were included.

Behavioral data. We used total cognitive scores, which were calculated from fluid and crystallized composite scores within the NIH Toolbox by the ABCD team.[5] Uncorrected standard fluid composite scores were computed using the pattern comparison processing speed, list-sorting working memory, picture sequence memory, Flanker, and dimensional change card sort tests. Uncorrected standard

© 2023 Tomasi D et al. *JAMA Network Open.*

crystallized composite scores were calculated using the oral reading recognition and picture vocabulary tests

MRI data. The ABCD imaging protocols were harmonized for 3T MRI scanners (Siemens Prisma, Phillips, and General Electric 750 scanners) equipped with adult-size multi-channel coils capable of multiband echo planar imaging (EPI) at 21 sites; details are described elsewhere.[6,7] Briefly, 3D T1w inversion prepared RF-spoiled gradient echo and T2w variable flip angle fast spin echo pulse sequences were used for structural MRI with 1mm isotropic resolution. Functional MRI (fMRI) data were collected using T2*-weighted multiband echo planar imaging (EPI; TE/TR=30/800 ms, 2.4 mm isotropic resolution, flip angle=52 degree, 60 slices covering the entire brain, multiband slice acceleration=6).[6] Diffusion MRI data with 1.7mm isotropic resolution were acquired using multiband EPI[8,9] with slice acceleration factor = 3, five b-values (b = 0, 500, 1000, 2000, and 3000 s/mm^2), and 96 diffusion directions.[7] All major white matter tracts in the ABCD 2.0 data release were labeled using a probabilistic approach for automated segmentation of white matter fiber tracts[10] while excluding gray matter (GM) and cerebral spinal fluid (CSF) voxels.[7]

Quality Assurance. The automated QA procedures of the ABCD study are described elsewhere.[7] Furthermore, images were corrected for scanner-specific gradient distortions and intensity inhomogeneity. Trained raters inspected images for poor quality, and artifacts such as blurring, ghosting, or ringing that could prevent brain segmentation.[7]

Head motion. Motion censoring information estimated with the ABCD-BIDS pipeline was used to remove time frames with FD>0.5mm. Since head motion is also a concern for pediatric structural and functional neuroimaging,[11] we further controlled for the subjects' tendency to move their head while in the scanner, as informed by the subjects' average FD during resting-state fMRI scans.

ABCD-BIDS pipeline. Like the HCP pipeline, the ABCD-BIDS pipeline comprises 5 consecutive steps: *PreFreesurfer*, performs brain extraction, denoising, and normalization of structural data to a standard

© 2023 Tomasi D et al. *JAMA Network Open.*

template; *Freesurfer*, performs brain segmentation and creates cerebral surfaces with FreeSurfer,[7] which has been validated for use in children; [12] *PostFreesurfer*, converts brain surfaces into the HCP-compatible CIFTI format; *fMRIVolume*, registers the functional time series to the volumetric standard template; and *fMRISurface*, converts functional time series data to the CIFTI format. Differences between the HCP and ABCD-BIDS pipelines are fully described elsewhere.[13] Briefly, the ABCD-BIDS pipeline does not require T2w images and performs the nonlinear registration to the standard atlas in *PostFreeSurfer*, which increases the effectiveness of the registration. Additionally, the ABCD-BIDS pipeline uses ANTS[14] for nonlinear registration which consistently outperforms other nonlinear registration methods.[15] In addition, the *fMRISurface* step in the ABCD-BIDS pipeline includes functional connectivity pre-processing that separates true head motion from fictitious motion induced by breathing-related magnetic field changes, [16] and performs standard denoising by regressing out time-varying head motion, white matter and CSF signals, and the global signals that may impact group comparisons.[17,18]

ABCC's "matched group" status. To maximize the relative independence of the Discovery and Replication samples, family members were kept together in the same sample and the samples were matched to have equivalent numbers of sibling and twin pairs and triplets.

Global FCD. Two grayordinates were considered functionally connected if their time-varying signals had a correlation R>0.6; this arbitrary correlation threshold was selected to be consistent with the threshold used by FCD mapping.[19] The gFCD (also called degree) at a given grayordinate, x0, was computed as the logarithm of the total number of functional connections (edges of the adjacency matrix), k(x0), between x0 and all other grayordinates in the brain. This calculation was repeated for all x0 grayordinates in the brain involving the computation of a correlation matrix with 91,282x91,282 elements. gFCD was estimated with Matlab 2017b (MathWorks, inc., Natick, MA). The average gFCD pattern across children (eFig 1) resembled that of adults.[20] Children demonstrated strong connectivity hubs in the posterior cingulate cortex (PCC), occipital, motor, and inferior parietal regions, which orchestrate the resting-state

© 2023 Tomasi D et al. *JAMA Network Open.*

networks (RSN) in the adult brain[21], and subcortical regions had lower gFCD (except for the hubs in the posterior cerebellar lobe). The high correlation across grayordinates of the gFCD in the *Discovery* and *Replication* samples (R=0.998) demonstrated the high reproducibility of this pattern (eFig 2).

Graphical tools. The Connectome Workbench (https://www.humanconnectome.org) and RStudio (https://www.rstudio.com/) were used for the visualization of CIFTI data and to create the figures.

© 2023 Tomasi D et al. *JAMA Network Open.*

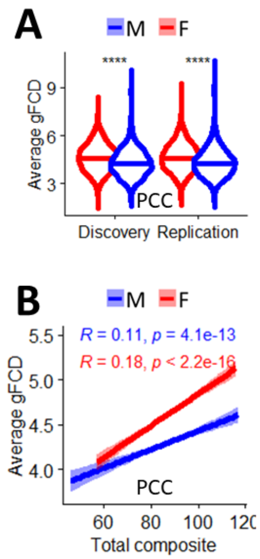

eFigure 1. Global Functional Connectivity Density (gFCD) In the Posterior Cingulate Cortex (PCC): Reproducibility and Association With Cognition.

The sex differences in gFCD were strongest in the PCC (A) and higher gFCD was associated with higher total cognition scores (B) in 4247 females (F) and 4492 males (M). Statistical model: ANCOVA. **** p <= 0.0001.

© 2023 Tomasi D et al. *JAMA Network Open.*

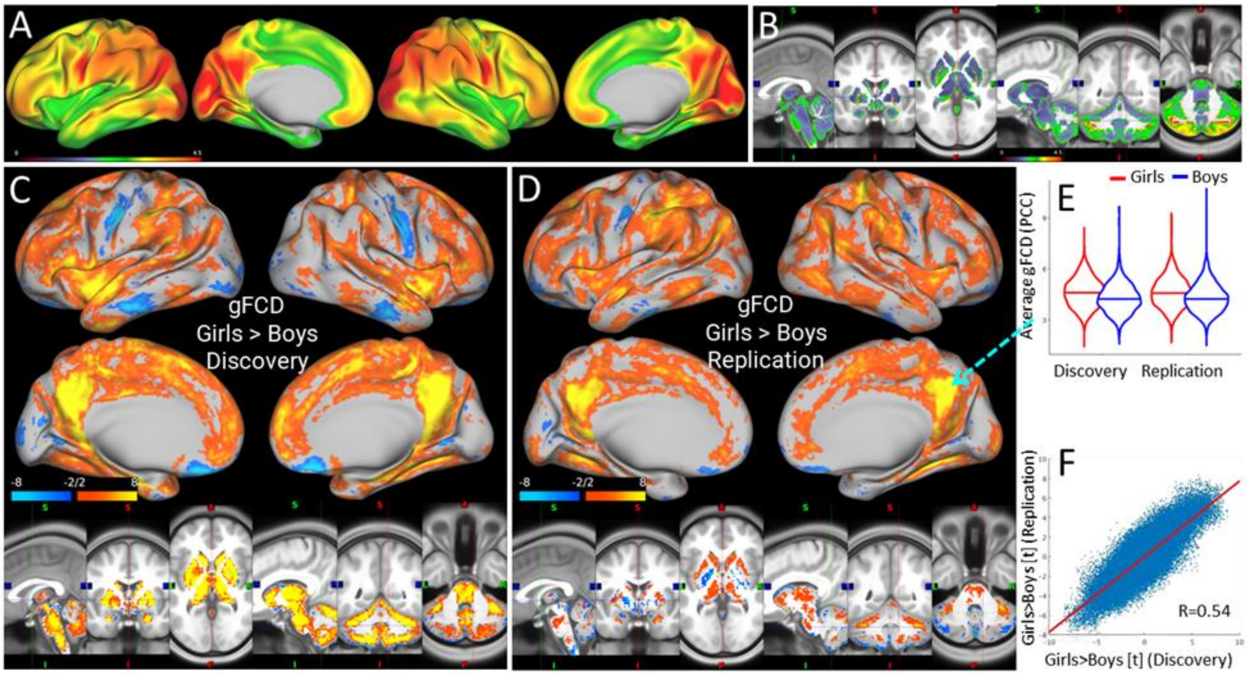

eFigure 2. Reproducibility of Sex Differences in gFCD

Surface renderings (**A**) and two sets of orthogonal views (**B**) depict the average distribution of global functional connectivity density (gFCD) in the brain of ABCD children. T-score maps overlaid on surfaces and orthogonal views of a template brain, with an FDR-corrected threshold P<0.05, showing the main effect of sex on gFCD, which was observed in the *Discovery* (**C**; 2181 girls and 2224 boys) and *Replication* (**D**; 2066 girls and 2268 boys) samples. The sex differences in gFCD were strongest in the posterior cingulum (PCC; **E**). The correlation across 91,282 grayordinates between t-scores in *Discovery* and *Replication* samples highlights the reproducibility of the sex differences in gFCD (**F**). Statistical model: ANCOVA.

© 2023 Tomasi D et al. *JAMA Network Open.*

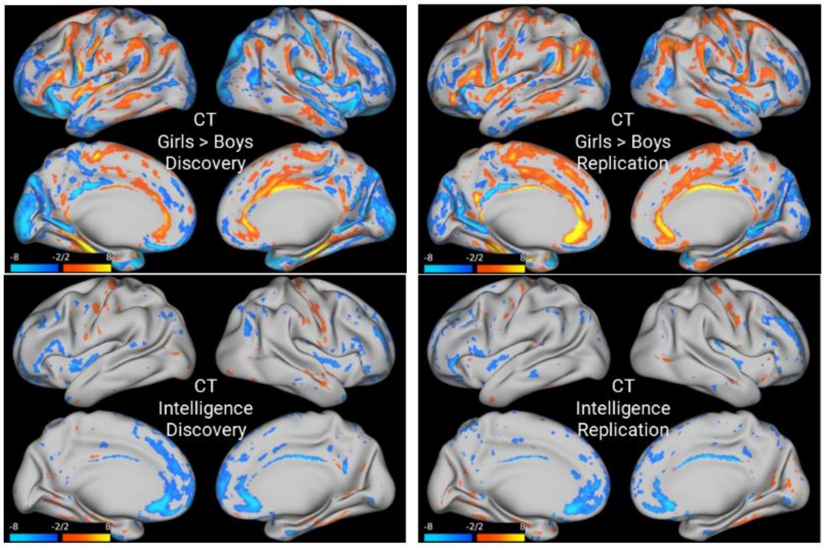

eFigure 3. Effects of Sex and Total Cognition on Cortical Thickness

Brain surface overlaid showing the main effects of sex and total cognition on cortical thickness (CT) observed in the *Discovery* (**A**; 2181 girls and 2224 boys) and *Replication* (**B**; 2066 girls and 2268 boys) samples. Statistical model: ANCOVA; FDR-corrected.

© 2023 Tomasi D et al. *JAMA Network Open.*

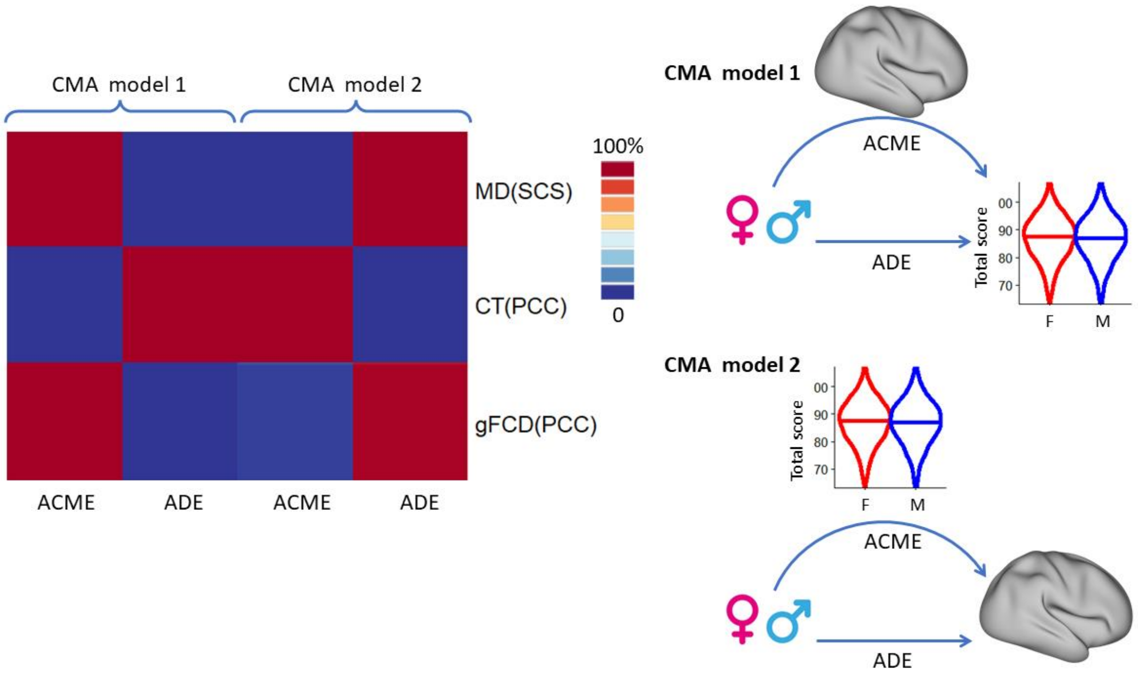

eFigure 4. Causal Mediation Analysis (CMA)

Significant proportions of direct (ADE) and causal mediation (ACME) effects for 2 different CMA models linking brain metrics [mean diffusivity in right superior corticostriatal bundle, MD(SCS), as well as cortical thickness, CT(PCC), and gFCD(PCC) in left posterior cingulate cortex (PCC)], sex, and total cognition scores in ABCD children. Sample: 4,247 females and 4,492 males for CT and gFCD; 2,779 females, F, and 3,018 males, M, for MD. P<0.05, Bonferroni corrected for multiple comparisons.

© 2023 Tomasi D et al. *JAMA Network Open.*

eTable. Effects of Sex, Total Composite Score, and Testosterone on Average Values of Global Functional Connectivity Density (gFCD) and Cortical Thickness (CT) in the Posterior Cingulum Seed, and on Average Values of Mean Diffusivity (MD) in the Right Cortico-Striatal Fiber Bundle

	**F-val**	**p-val**	**partial η^2**
Model 1: gFCD ~ sex*total + Age			
**Sex**	308.6	<2E-16	0.04
**Total**	174.2	<2E-16	0.02
**Sex:total**	15.9	1.6E-04	0.002
**Age**	0.1	0.73	0
Model 2: MD~ sex*total + Age			
**Sex**	120.2	<2E-16	0.02
**Total**	105.1	<2E-06	0.02
**Sex:Total**	1.08	0.3	0
**Age**	198.2	<2E-16	0.04
Model 3: CT~ sex*total + Age			
**Sex**	25.3	5.1E-07	0.003
**Total**	21.9	2.8E-06	0.003
**Sex:Total**	1.81	0.18	0
**Age**	25.8	3.8E-07	0.003

Statistical model: ANCOVA. Sample size for Models 1 and 3: 4,492 boys and 4,247 girls; for Model 2: 3,018 boys and 2,779 girls with Siemens MRI data.

© 2023 Tomasi D et al. *JAMA Network Open.*

eReferences

1. Garavan H, Bartsch H, Conway K, et al. Recruiting the ABCD sample: Design considerations and procedures. *Dev Cogn Neurosci.* 2018;32:16-22.
2. Thompson W, Barch D, Bjork J, et al. The structure of cognition in 9 and 10 year-old children and associations with problem behaviors: Findings from the ABCD study's baseline neurocognitive battery. *Dev Cogn Neurosci.* 2019;36:100606.
3. Jernigan T, Brown S. Introduction. *Dev Cogn Neurosci.* 2018;32:1-3.
4. Karcher N, Barch D, Avenevoli S, et al. Assessment of the Prodromal Questionnaire-Brief Child Version for Measurement of Self-reported Psychoticlike Experiences in Childhood. *JAMA Psychiatry.* 2018;75(8):853-861.
5. Luciana M, Bjork J, Nagel B, et al. Adolescent neurocognitive development and impacts of substance use: Overview of the adolescent brain cognitive development (ABCD) baseline neurocognition battery. *Dev Cogn Neurosci.* 2018;32:67-79.
6. Casey B, Cannonier T, Conley M, et al. The adolescent brain cognitive development (ABCD) study: imaging acquisition across 21 sites. *Dev Cogn Neurosci.* 2018;32:43-54.
7. Hagler DJ, Hatton S, Cornejo M, et al. Image processing and analysis methods for the Adolescent Brain Cognitive Development Study. *Neuroimage.* 2019;202:116091.
8. Moeller S, Yacoub E, Olman C, et al. Multiband multislice GE-EPI at 7 tesla, with 16-fold acceleration using partial parallel imaging with application to high spatial and temporal whole-brain fMRI. *Magn Reson Med.* 2010;63(5):1144-1153.
9. Setsompop K, Gagoski B, Polimeni J, Witzel T, Wedeen V, Wald L. Blipped-controlled aliasing in parallel imaging for simultaneous multislice echo planar imaging with reduced g-factor penalty. *Magn Reson Med.* 2012;67(5):1210-1224.
10. Hagler DJ, Ahmadi M, Kuperman J, et al. Automated white-matter tractography using a probabilistic diffusion tensor atlas: Application to temporal lobe epilepsy. *Hum Brain Mapp.* 2009;30:1535-1547.
11. Power J, Barnes K, Snyder A, Schlaggar B, Petersen S. Spurious but systematic correlations in functional connectivity MRI networks arise from subject motion. *Neuroimage.* 2012;59(3):2142-2154.
12. Ghosh S, Kakunoori S, Augustinack J, et al. Evaluating the validity of volume-based and surface-based brain image registration for developmental cognitive neuroscience studies in children 4 to 11 years of age. *Neuroimage.* 2010;53(1):85-93.
13. Feczko E, Conan G, Marek S, et al. Adolescent Brain Cognitive Development (ABCD) Community MRI Collection and Utilities. *bioRxiv.*doi: https://doi.org/10.1101/2021.07.09.451638.
14. Avants B, Epstein C, Grossman M, Gee J. Symmetric diffeomorphic image registration with cross-correlation: Evaluating automated labeling of elderly and neurodegenerative brain. *Med Image Anal.* 2008;12(1):26-41.
15. Ou Y, Akbari H, Bilello M, Da X, Davatzikos C. Evaluation of Registration Algorithms in Different Brain Databases With Varying Difficulty: Results and Insights. *IEEE Trans Med Imaging.* 2014;33(10):2039-2065.
16. Fair D, Miranda-Dominguez O, Snyder A, et al. Correction of respiratory artifacts in MRI head motion estimates. *Neuroimage.* 2020;208:116400.
17. Ciric R, Wolf D, Power J, et al. Benchmarking of participant-level confound regression strategies for the control of motion artifact in studies of functional connectivity. *Neuroimage.* 2017;154:174-187.
18. Power J, Schlaggar B, Petersen S. Recent progress and outstanding issues in motion correction in resting state fMRI. *Neuroimage.* 2015;105(536:551).

© 2023 Tomasi D et al. *JAMA Network Open.*

19. Tomasi D, Volkow N. Functional Connectivity Density Mapping. *Proceedings of the National Academy of Sciences U S A.* 2010;107(21):9885-9890.
20. Tomasi D, Volkow N. Functional connectivity hubs in the human brain. *Neuroimage.* 2011;57(3):908-917.
21. Tomasi D, Volkow N. Association between Functional Connectivity Hubs and Brain Networks. *Cereb Cortex.* 2011;21(9):2003-2013.

© 2023 Tomasi D et al. *JAMA Network Open.*
