# Supplementary material for: Measures of Brain Connectivity and Cognition by Sex in US Children
Source: JAMA Netw Open. 2023 Feb 21;6(2):e230157. doi: 10.1001/jamanetworkopen.2023.0157 (PMC9945095; doi:10.1001/jamanetworkopen.2023.0157)
Supplement: Supplement 2. — Data Sharing Statement [file jamanetwopen-e230157-s002.pdf]

Data Sharing Statement

Tomasi. Measures of Brain Connectivity and Cognition by Sex in US Children. *JAMA Netw Open*. Published February 21, 2023. doi:10.1001/jamanetworkopen.2023.0157

Data
Data available: No

Additional Information
Explanation for why data not available: The data was made available already by the ABCD study https://nda.nih.gov/
